# Mortality and revision rate of cemented and uncemented hemiarthroplasty after hip fracture: an analysis of the Dutch Arthroplasty Register (LROI)

**DOI:** 10.1080/17453674.2020.1752522

**Published:** 2020-04-14

**Authors:** Bouke J Duijnisveld, Koen L M Koenraadt, Liza N van Steenbergen, Stefan B T Bolder

**Affiliations:** a Department of Orthopaedic Surgery, Sint Maartenskliniek, Nijmegen;; b Foundation for Orthopaedic Research, Care and Education, Amphia Hospital, Breda;; c Dutch Arthroplasty Register, Landelijke Registratie Orthopedische Implantaten (LROI), ‘s Hertogenbosch;; d Department of Orthopaedic Surgery, Amphia Hospital, Breda, The Netherlands

## Abstract

Background and purpose — Femoral neck fractures are commonly treated with cemented or uncemented hemiarthroplasties (HA). We evaluated differences in mortality and revision rates in this fragile patient group.

Patients and methods — From January 1, 2007 until December 31, 2016, 22,356 HA procedures from the Dutch Arthroplasty Register (LROI) were included. For each HA, follow-up until death, revision, or end of follow-up (December 31, 2016) was determined. The crude revision rate was determined by competing risk analysis. Multivariable Cox regression analyses were performed to evaluate the effect of fixation method (cemented vs. uncemented) on death or revision. Age, sex, BMI, Orthopaedic Data Evaluation Panel (ODEP) rating, ASA grade, surgical approach, and previous surgery were included as potential confounders.

Results — 1-year mortality rates did not differ between cemented and uncemented HA. 9-year mortality rates were 53% (95% CI 52–54) in cemented HA compared to 56% (CI 54–58) in uncemented HA. Multivariable Cox regression analysis showed similar mortality between cemented and uncemented HA (HR 1.0, CI 0.96–1.1). A statistically significantly lower 9-year revision rate of 3.1% (CI 2.7–3.6) in cemented HA compared with 5.1% (CI 4.2–6.2) in the uncemented HA was found with a lower hazard ratio for revision in cemented compared with uncemented HA (HR 0.56, CI 0.47–0.67).

Interpretation — Long-term mortality rates did not differ between patients with a cemented or uncemented HA after an acute femoral neck fracture. Revision rates were lower in cemented compared with uncemented HA.

The number of hemiarthroplasties (HA) after displaced femoral neck fracture increases as a result of global aging, and inferior results and high risk of reoperation after internal fixation. Although the literature on the decision to use cemented or uncemented HA may favor a cemented implant, both techniques are currently used. The use of bone cement is associated with bone cement implantation syndrome (BCIS) characterized by hypoxia, hypotension, loss of consciousness around the time of bone cementation, and intraoperative death (Olsen et al. 2014, Rutter et al. 2014). More intraoperative complications including intraoperative death were found in cemented HA in the Norwegian register (Gjertsen et al. 2012, Talsnes et al. 2013). However, no differences in mortality were found after 1 week (Costain et al. 2011, Yli-Kyyny et al. 2014). More studies including randomized controlled trials (Deangelis et al. 2012, Taylor et al. 2012) and registry studies (Costa et al. 2011, Ekman et al. 2019) did not show differences in mortality between cemented and uncemented HA. Randomized controlled trials (Taylor et al. 2012, Langslet et al. 2014, Inngul et al. 2015) and register studies (Gjertsen et al. 2012, Yli-Kyyny et al. 2014) have shown that the use of uncemented implants could result in a higher risk of periprosthetic fractures. A meta-analysis by Li et al. (2013) concluded that differences in several outcome parameters indicated cemented hemiarthroplasty to be superior to the uncemented counterpart. However, a serious flaw in this analysis is that several studies were included using an outdated stem like the Austin Moore (Sonne-Holm et al. 1982, Emery et al. 1991, Parker et al. 2010) and the experimental uncemented Thomson stem (Sadr and Arden 1977). The use of a prosthesis without Orthopaedic Data Evaluation Panel (ODEP) rating > 3A could influence outcome and is therefore discouraged (Grammatopoulos et al. 2015). A recent review by Rogmark and Leonardsson (2016) included 5 randomized studies comparing modern uncemented and cemented hemiarthroplasties. They found no differences in mortality, but more periprosthetic fractures in uncemented cases. We compared cemented and uncemented HA after an acute hip fracture with primary outcome mortality and revision rate. Data from the Dutch Arthroplasty Register (LROI) were used and the cohort of cemented HAs was compared with uncemented HAs, accounting for the ODEP rating and other confounders.

## Patients and methods

The Dutch Arthroplasty Register (LROI) is a nationwide population-based register that includes information on arthroplasties in the Netherlands since 2007. It covers 100% of Dutch hospitals and has a completeness of reporting of 70% for primary orthopedic HAs in 2013 to 88% in 2016 (Van Steenbergen et al. 2015).

The LROI database contains information on patient, procedure, and prosthesis characteristics registered by registrars from each hospital. For each component a product number is registered to identify the characteristics of the prosthesis. Vital status of all patients was obtained actively on a regular basis from Vektis, the national insurance database on health care in the Netherlands, which records all deaths of Dutch citizens. The LROI requires the informed consent of patients and uses an opt-out system in this respect.

For this study, we included all HA procedures in patients with an acute femoral neck fracture registered by orthopedic surgeons in the LROI from January 1, 2007 until December 31, 2016 (N = 22,351). All femoral stems were classified as ODEP rating 3A or other/no rating by checking the prosthesis in the ODEP database (http://www.odep.org.uk). Prostheses that were not available in the ODEP database were manually explored by the researchers. When these prostheses were not found as ODEP rating ≥ 3A, they were classified as “no ODEP 3A rating.” Other parameters such as age, sex, BMI, ASA classification, and previous surgery on the affected hip were used from the LROI database. BMI and smoking have only been available in the LROI since 2014. Closed reductions after a dislocation or incision and drainage for infection were not included in the LROI, as in these procedures no component exchange was performed. The median follow-up was 1.8 years (0–10).

### Statistics

Kaplan–Meier survival analysis was performed to examine the survival rates of the patients over time. A multivariable Cox proportional hazard analysis was performed to examine the effect of fixation type (i.e., cemented vs. uncemented) on death after HA. Demographic variables such as age, sex, ASA classification, BMI, and smoking habit were included as covariates. Age and BMI with impossible values were excluded.

Survival time for revision was calculated as the time from primary HA to the first revision arthroplasty for any reason, death of the patient, or the end of the study follow-up (December 31, 2016). Cumulative crude incidence of revision was calculated using competing risk analysis where death was considered to be the competing event (Lacny et al. 2015, Wongworawat et al. 2015). Multivariable Cox proportional hazard analyses were performed to examine the effect of fixation type on revision. Demographic variables and possible risk factors including approach, ODEP rating (> 3A vs. other/no rating), and previous surgery on the affected hip were included as covariates.

Furthermore, the reasons for revision were compared between cemented and uncemented HA. Finally, multivariable logistic regression analyses were performed on the revisions (n = 517) to examine independent risk factors for revision due to dislocation, infection, femoral loosening, periprosthetic fracture, or other reasons for revision. Demographic variables and possible risk factors were included as covariates in the multivariable logistic regression analysis. All confidence intervals (CI) are defined as 95%. For the CI, we assumed that the number of observed cases followed a Poisson distribution.

### Ethics, funding, and potential conflicts of interest

Ethical approval was not required for this study. Our Foundation for Orthopedic Research, Care and Education (FORCE) receives money from Zimmer-Biomet, Stryker, and Mathys not directly related to this study. SB is a consultant for Stryker.

## Results

Patients and procedure characteristics are given in Table 1.

**Table 1.** Patient characteristics of cemented (n = 14,736) and uncemented (n = 7,615) hemiarthroplasties in the Netherlands 2007–2016. Values are number (%) unless otherwise specified

Factor	Cemented hemiarthroplasty n = 14,736	Uncemented hemiarthroplasty n = 7,615
Mean age (SD)	82.5 (8.1)	82.7 (7.9)
Sex		
Male	4,345 (30)	2,271 (30)
Female	10,362 (70)	5,317 (70)
Missing	29 (0.2)	27 (0.4)
ASA classification		
I	334 (2)	172 (2)
II	5,514 (37)	2,730 (36)
III or IV	8,490 (58)	4,540 (60)
Missing	398 (3)	173 (2)
Previous surgery on affected joint		
Yes	147 (1)	71 (1)
No	13,858 (94)	6,995 (92)
Missing	575 (4)	480 (6)
Smoking ^a^		
Yes	526 (4)	193 (3)
No	5,370 (36)	2,603 (34)
Missing	8,840 (60)	4,819 (63)
Surgical approach		
Posterolateral	7,639 (52)	4,098 (54)
Direct lateral	5,149 (35)	2,330 (31)
Anterolateral	1,646 (11)	884 (12)
Anterior	172 (1)	230 (3)
Other or missing	117 (1)	60 (1)
ODEP 3A rating		
Yes	13,189 (90)	5,078 (67)
No	1,091 (7)	2,334 (31)
Missing	203 (3)	456 (3)
Mean BMI (SD) ^a^	24.3 (4.2)	23.6 (5.1)

BMI: body mass index, ASA: American Society of Anesthesiologists, ODEP: Orthopaedic Data Evaluation Panel.

**^a^**Only available for the period 2014–2016.

### Mortality

**Figure 1.**
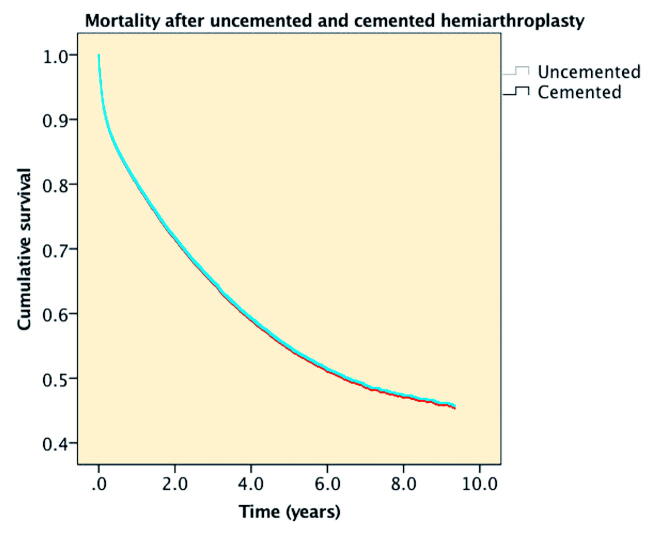
Cumulative mortality rate of uncemented (n = 7,615) and cemented (n = 14,736) hemiarthroplasties in the Netherlands 2007–2016.

1-week mortality was 2.1% (CI 1.8–2.3) in the cemented HA group compared with 1.8% (CI 1.6–2.2) in the uncemented HA group. 1-month mortality was 6.0% (CI 5.6–6.4) in the cemented HA group compared with 5.4% (CI 4.9–6.0) in the uncemented HA group. 1-year mortality was 19.7% (CI 19.1–20.4) in the cemented HA group compared with 19.5% (CI 18.6–20.4) in the uncemented HA group. 9-year mortality rates were 53% (CI 52–54) in the cemented HA group compared with 56% (CI 54–58) in the uncemented HA group (Figure 1). Univariable Cox regression analysis showed no statistically significant difference in mortality between patients with a cemented and an uncemented HA (HR 0.99, CI 0.95–1.04). Multivariable Cox regression analysis showed an HR of 1.00 (CI 0.96–1.05) adjusted for age at surgery, sex, and ASA classification. In a subset from 2014 to 2016 that included also BMI and smoking, no statistically significant difference in mortality in cemented and uncemented HA was found (HR 1.06, CI 0.97–1.15).

### Revision

**Figure 2.**
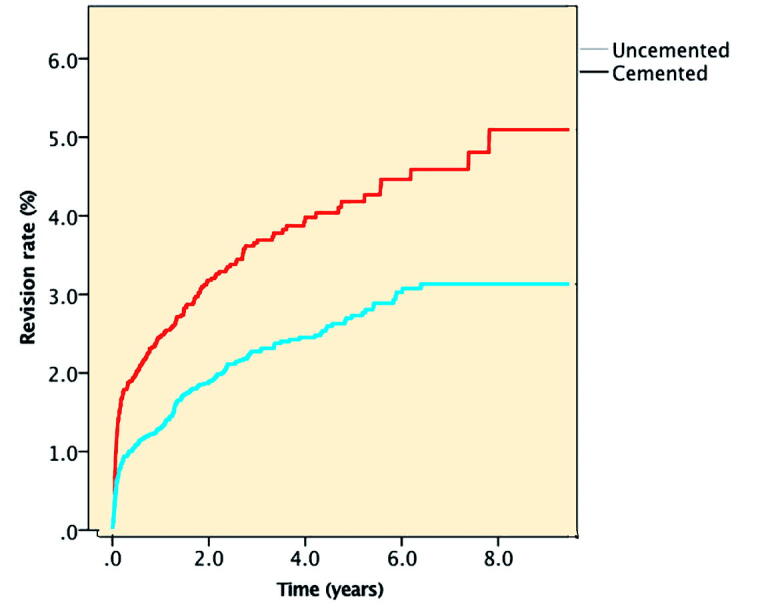
Crude cumulative revision rate of uncemented (n = 7,615) and cemented (n = 14,736) hemiarthroplasties in the Netherlands 2007–2016.

Competing risk analysis showed a lower crude revision rate at 1 month (0.5%, CI 0.4–0.7), 1 year (1.3%, CI 1.1–1.5), and 9 years (3.1%, CI 2.7–3.6) in cemented HA compared with the revision rate at 1 month (1.1%, CI 0.9–1.4), 1 year (2.5%, CI 2.1–2.9), and 9 years (5.1%, CI 4.2–6.2) in uncemented HA (Figure 2). Multivariable Cox regression revealed a lower hazard ratio for revision (HR 0.56, CI 0.47–0.67) in cemented compared with uncemented HA, adjusted for confounders including sex, age, ASA classification, approach, ODEP rating, and previous surgery on the affected hip. These findings were persistent after adjusting also for BMI and smoking in the subset from 2014 to 2016.

### Reasons for revision

Dislocation, infection, femoral loosening, and periprosthetic fractures were the most common reason for revision of HA (Table 2). Multivariable logistic regression analysis showed that the risk for dislocation was lower in direct lateral approach (HR 0.37, CI 0.24–0.56) and anterolateral approach (HR 0.32, CI 0.16–0.66) compared with posterolateral approach.

**Table 2.** Reasons for revision of cemented and uncemented hemiarthroplasty

Reason	Cemented n = 14,726 n (%)	Uncemented n = 7,551 n (%)	Hazard **^a^** ratio (95% CI)	p-value
Dislocation	92 (0.6)	62 (0.8)	0.77 (0.55–1.08)	0.1
Infection	55 (0.4)	19 (0.2)	1.48 (0.85–2.56)	0.2
Loosening of stem	19 (0.1)	40 (0.5)	0.21 (0.12–0.36)	< 0.001
Periprosthetic fracture	12 (0.1)	77 (1.0)	0.07 (0.04–0.13)	< 0.001
Other	105 (0.7)	78 (1.0)	0.66 (0.48–0.90)	0.009

**^a^**Hazard ratios with 95% CI and p-values are shown for logistic regression analysis for fixation type, adjusted for age, sex, ASA classification, previous surgery, surgical approach, and ODEP rating.

Risk for revision because of infection was statistically significantly higher in patients with previous surgery (HR 4.0, CI 1.3–13). Femoral stem loosening was less often the reason for revision in the cemented HA group compared with the uncemented HA group (HR 0.21, CI 0.12–0.36) and more often in patients aged 60– 80 compared with those aged > 80 years (HR 2.2, CI 1.3–3.8). The risk for revision because of a periprosthetic fracture was less in cemented HA compared with cemented HA (HR 0.07, CI 0.04–0.13).

## Discussion

In this study with more than 22,000 hemiarthroplasties for acute femoral neck fracture from the Dutch Arthroplasty Register, we found comparable 9-year mortality rates between cemented and uncemented HA. The 9-year revision rate was lower in cemented HA compared with uncemented HA. Dislocation, infection, femoral stem loosening, and periprosthetic fractures were the most common reasons for revision.

The register data show that in the Netherlands one-third of hemiarthroplasties are performed with an uncemented stem. One of the reasons for choosing an uncemented stem may be the assumed risk for BCIS when using a cemented stem in the fragile patient. We found a difference in early mortality rates in favor of the uncemented group. This might indicate the presence of BCIS, although this cannot be proven from a register study and could also be the result of selection bias. Our 1-month mortality rate was lower in the uncemented HA group. However, the difference was small and the 1-month mortality rate in the cemented HA was comparable to mortality rates of 5–8% previously found in the literature (Costain et al. 2011, Olsen et al. 2014). From these results, in patients with high risk for BCIS, an uncemented HA may be a good option to improve the earliest outcome. Olsen et al. (2014) showed an incidence of 21%, 5.1%, and 1.7% of BCIS grades 1, 2, and 3 respectively with a 1-month mortality of 9.3%, 35%, and 88% respectively.

BCIS could also influence morbidity due to hypoxia and hypotension leading to a higher mortality during follow-up. An increased mortality in the cemented HA group was, however, not observed compared with the uncemented HA group at 1- and 9-year follow-up. This effect may be due to a higher revision rate in the uncemented group. Our 1-year mortality rate of 20% is comparable to results from the Norwegian and the Swedish Registry (Leonardsson et al. 2012, Gjertsen et al. 2017). Our 9-year mortality rates of 53% and 56% are in line with the mortality rate of 45% found in the Swedish Registry after 7 years’ follow-up (Jawad et al. 2019). A recent study of the Norway Registry showed a higher mortality of about 90% at 9 years’ follow-up (Kristensen et al. 2019). This could be due to patient selection, as Kristensen et al. selected only patients of 70 years of age or older, whereas in the current study and in the study of the Swedish Registry no age selection was performed.

Our findings of a lower revision rate after cemented HA, compared with uncemented HA, is supported by other register studies (Gjertsen et al. 2012, Jameson et al. 2013, Yli-Kyyny et al. 2014, Kristensen et al. 2019). We have no data on reoperations other than revision procedures in the Dutch register. Other studies showed a reoperation rate of 7–11% at 5–19 years’ follow-up (Parker et al. 2010, Viberg et al. 2013). These reoperation rates cannot be compared with our revision rates as reoperations do not always include a revision. Surprisingly, ODEP rating > 3A did not influence revision rate in our study. Although individual prosthesis brands could in theory influence revision rate, the data on individual prosthesis brands were not available for this study.

Dislocation, infection, femoral stem loosening, and periprosthetic fractures were the most common reasons for revision. The posterolateral approach was an independent risk factor for dislocation as shown earlier (Leonardsson et al. 2012, Rogmark et al. 2014, Moerman et al. 2018). The posterolateral approach could be considered as surgical approach in HA because functional outcome including pain, walking without mobility aids, and patient-reported outcome measures have been shown to be in favor of the posterolateral approach (Kristensen et al. 2017, Hongisto et al. 2018). We could not measure the functional outcome. Mukka et al. (2017) did not find any differences in functional outcome between direct lateral and posterolateral approach. We found an uncemented HA as independent risk factor for femoral loosening and periprosthetic fracture as previously shown (Leonardsson et al. 2012, Rogmark et al. 2014, Moerman et al. 2018), which could also be influenced by a lower threshold to revise an uncemented stem when compared with a cemented implant.

The strength of this population-based registry study is the large study population of over 22,000 patients with follow-up of up to 9 years and the inclusion of several potential confounders such as patient, procedure, and prosthesis characteristics, like type of fixation and ODEP rating. A limitation of our study is the observational nature of the data. Therefore, causal relationships cannot be identified. To minimize selection bias, confounders including age, sex, ASA classification, BMI, previous surgery, and smoking habit were added to the multivariable regression analysis. However, potential residual confounding like socioeconomic factors and alcohol consumption could still be present. Severely ill elderly patients could have received internal fixation or non-operative treatment instead of HA. Also, “young old” patients could receive HA rather than THA or internal fixation. Both regimes would lower the mortality after HA. Furthermore, the Dutch Arthroplasty Register does not allow for comparison of individual prosthesis brands. Reoperations like debridement of the wound or Vancouver B1 fracture fixation without prosthesis component replacement are not registered in the Dutch Arthroplasty Register. This may influence the view on fracture rates in our cohort. In addition, closed reduction for a dislocated hip and acetabular erosion as reason for revision are not registered in the LROI database and are therefore not included in this study. Because of these reoperations without revision of the prosthesis, reoperation rate will be higher than revision rate.

An uncemented HA may be considered for the patient with high risk for BCIS with short life expectancy. Regular use of an uncemented stem does not seem to offer benefit to patients. We found no evidence that cemented HA leads to higher mortality in the longer term. In summary, based on the outcome of this study and earlier findings in the literature, in which long-term mortality rates were similar between cemented and uncemented HA for displaced femoral neck fracture and revision rates were lower in cemented HA, we recommend the use of a cemented HA for patients with an acute femoral neck fracture.
